# Supplementary material for: Timing is everything: Early do-not-resuscitate orders in the intensive care unit and patient outcomes
Source: PLoS One. 2020 Feb 18;15(2):e0227971. doi: 10.1371/journal.pone.0227971 (PMC7028295; doi:10.1371/journal.pone.0227971)
Supplement: S2 File — (PDF) [file pone.0227971.s002.pdf]

A MULTI-INSTITUTIONAL CONSORTIUM AT WEILL CORNELL MEDICAL COLLEGE WITH: Cornell University, Ith
Cornell University Cooperative Extension, New York City / Hospital for Special Surgery / Hunter College School of Nur
Hunter Center for Study of Gene Structure and Function / Memorial Sloan-Kettering Cancer Center / NewY
Presbyterian Hospital/Weill Cornell Medical Center / Weill Cornell Graduate School of Medical Sciences

Weill Cornell Medical College
Clinical and Translational Science Center

Quality of Life in the Last Week of Life in the ICU

Codebook

Data Dictionary Codebook

02/27/2017 11:55am

#	Variable / Field Name	Field Label<br>*Field Note*	Field Attributes (Field Type, Validation, Choices, Calculations, etc.)
Instrument: **Postmortem Assessment**			
1	record_id	Record ID	text
2	info	BASIC INFORMATION OBTAINED BY STAFF	descriptive
3	nurse_id	Nurse ID	text
4	total_shifts	Number of total shifts with patient	text
5	day_shifts	Number of day shifts with patient	text (integer)
6	night_shifts	Number of night shifts with patient	text (integer)
7	present_at_death	Present at patient's death?	yesno<br>1 Yes<br>0 No
8	time_shift_death<br><br>Show the field ONLY if:<br>[present_at_death] = '0'	Number of days between last shift and patient's death	text
9	time_death_interview	Number of days between patient's death and interview	text
10	patient_id	Patient ID	text
11	gender	Gender	radio<br>1 Male<br>2 Female

12	race	Race/Ethnicity	checkbox<br>1 race___1 White<br>2 race___2 Black or African American<br>4 race___4 Asian/Pacific Islander<br>5 race___5 Native American or American Indian<br>6 race___6 More than one race<br>3 race___3 Hispanic or Latino<br>7 race___7 Non-Hispanic or Non-Latino
13	age	Age	text (number)
14	cause_of_death	Cause of Death	text
15	location_of_death	Location of Death	radio<br>1 Hospital - ICU<br>2 Hospital - not ICU
16	location<br>Show the field ONLY if: [location_of_death] = '2'	Location of death if not ICU	text
17	length_of_stay	Length of Stay in ICU (days)	text (integer)
18	hospital_stay	Length of stay in hospital (days)	text
19	diagnoses	Admitting Diagnosis	notes
20	medical_care	Medical care received in the last week of life	checkbox<br>1 medical_care___1 Chemotherapy<br>2 medical_care___2 Vasopressors<br>3 medical_care___3 Dialysis<br>4 medical_care___4 Mechanical ventilation<br>5 medical_care___5 Feeding tube<br>6 medical_care___6 Cardiac resuscitation<br>7 medical_care___7 Surgical procedure<br>8 medical_care___8 Withdrawal of life sustaining therapy
21	meds	Other Medications	notes
22	scan_blood_other	Other tests (Scans, Blood, Other)	text

23	charlson	Charlson Index of Comorbidity (CIC)<br>Assigned Weight of 1:<br>Myocardial infarction<br>Congestive heart failure<br>Peripheral vascular disease<br>Cerebrovascular disease<br>Dementia<br>Chronic pulmonary disease<br>Connective tissue disease<br>Ulcer disease<br>Mild liver disease (without portal hypertension, inlcudes chronic hepatitis)<br>Diabetes without end-organ damage<br><br>Assigned weight of 2:<br>Hemiplegia<br>Moderate or severe renal disease<br>Diabetes with end organ damage<br>Any tumor<br>Leukemia<br>Lymphoma<br><br>Assigned weight of 3:<br>Moderate or severe liver disease<br><br>Assigned weight of 6:<br>Metastatic solid tumor<br>AIDS<br><br>Please list the conditions (of those cited above) that the patient suffers from.	notes
24	cic_total	Charleson score:<br>(Patients Age - 40)/10 + cormorbidity score =	text
25	rass	What was the patient's average RASS score during his/her last week of life?	text
26	pain_level	What was the patient's average pain assessment score during his/her last week of life?	text (number, Min: 0, Max: 10)
27	frequency_pain<br><br>Show the field ONLY if:<br>[pain_level] > 0	How often was the patient able to self-report pain?	text
28	urinary_incont	In the last week of the patient's life, did he/she have urinary incontinence?	yesno<br>1 | Yes<br>0 | No
29	incont_management	If yes, how was the patient's urinary incontinence managed?	checkbox<br>1 | incont_management___1 | Bathroom<br>2 | incont_management___2 | Commode<br>3 | incont_management___3 | Bedpan/urinal<br>4 | incont_management___4 | Diaper/absorp[illegible] product<br>5 | incont_management___5 | Urethral cathe[illegible]<br>6 | incont_management___6 | Condom cathe[illegible]<br>7 | incont_management___7 | Suprapubic catheter<br>8 | incont_management___8 | Other
30	incont_manage_other	Other	text

31	urinary_retention	In the last week of the patient's life, did he/she have urinary retention?	yesno<br>1 Yes<br>0 No
32	retention_management	If yes, how was this managed?	text
33	chaplain	Section Header: *In the patient's last week of life...*<br>was the patient visited by the hospital chaplain?	radio (Matrix)<br>1 No<br>2 Yes
34	palliative_consult	did the patient have a palliative care team consult?	radio (Matrix)<br>1 No<br>2 Yes
35	pall_care	If yes, was this in the ICU?	radio<br>1 Yes<br>0 No<br>Custom alignment: RH
36	advanced_directive	Advanced directive	yesno<br>1 Yes<br>0 No
37	dnr	DNR	yesno<br>1 Yes<br>0 No
38	postmortem	=======================POSTMORTEM ASSESSMENT=======================	descriptive
39	family_present	Section Header: *In the last week of the patient's life...*<br>were family and/or friends present?	radio (Matrix)<br>1 No<br>2 Yes
40	want_family_present	did he/she want family and/or friends to be there?	radio (Matrix)<br>1 No<br>2 Yes
41	goodbye	to the best of your knowledge, were there people the patient wanted to say goodbye to but couldn't?	radio (Matrix)<br>1 No<br>2 Yes
42	discharged	Was the patient scheduled to be discharged?	yesno<br>1 Yes<br>0 No
43	discharge_where<br>Show the field ONLY if: [discharged] = '1'	If yes, to where?	radio<br>1 Stepdown unit<br>2 General hospital floor<br>3 Hospice<br>4 Home<br>5 Rehab facility<br>6 Nursing home<br>7 Other

44	discharge_why<br><br>Show the field ONLY if:<br>[discharged] = '1'	If yes, why?	radio<br>1 | Patient preferred to die at home<br>2 | Patient preferred to die in hospice<br>3 | Insurance didn't cover extended hospital stay<br>4 | Patient's condition was improving/stabilizing
45	no_discharge<br><br>Show the field ONLY if:<br>[discharged] = '0'	If no, why?	radio<br>1 | Patient's medical condition made him/her ineligible<br>2 | Patient did not want to be discharged<br>3 | Attending physician did not want patient discharged<br>4 | Patient's family did not want patient discharged<br>5 | Waiting for space at another facility<br>6 | Other
46	not_discharged_other<br><br>Show the field ONLY if:<br>[no_discharge] = '6'	Other	text
47	communicating_effectively	In the last week of the patient's life, to what extent did he or she have difficulty communicating effectively?	radio<br>1 | 1 - No difficulty<br>2 | 2<br>3 | 3<br>4 | 4<br>5 | 5<br>6 | 6<br>7 | 7<br>8 | 8<br>9 | 9<br>10 | 10 - Extreme difficulty
48	how_pt_communicate	How did the patient communicate, if at all?	text
49	pt_asleep	Section Header: *In the last week of the patient's life...*<br>how often was he/she asleep/sedated?	radio (Matrix)<br>1 | 1 - Never/Not at all<br>2 | 2<br>3 | 3<br>4 | 4<br>5 | 5<br>6 | 6<br>7 | 7<br>8 | 8<br>9 | 9<br>10 | 10 - Always/Completely<br>11 | N/A - Unable to answer

<table>
<tr><td>50</td><td>pt_unconscious</td><td>how often was he/she unconscious?</td><td>radio (Matrix)<table><tr><td>1</td><td>1 - Never/Not at all</td></tr><tr><td>2</td><td>2</td></tr><tr><td>3</td><td>3</td></tr><tr><td>4</td><td>4</td></tr><tr><td>5</td><td>5</td></tr><tr><td>6</td><td>6</td></tr><tr><td>7</td><td>7</td></tr><tr><td>8</td><td>8</td></tr><tr><td>9</td><td>9</td></tr><tr><td>10</td><td>10 - Always/Completely</td></tr><tr><td>11</td><td>N/A - Unable to answer</td></tr></table></td></tr>
<tr><td>51</td><td>pt_confused</td><td>how often was he/she confused?</td><td>radio (Matrix)<table><tr><td>1</td><td>1 - Never/Not at all</td></tr><tr><td>2</td><td>2</td></tr><tr><td>3</td><td>3</td></tr><tr><td>4</td><td>4</td></tr><tr><td>5</td><td>5</td></tr><tr><td>6</td><td>6</td></tr><tr><td>7</td><td>7</td></tr><tr><td>8</td><td>8</td></tr><tr><td>9</td><td>9</td></tr><tr><td>10</td><td>10 - Always/Completely</td></tr><tr><td>11</td><td>N/A - Unable to answer</td></tr></table></td></tr>
<tr><td>52</td><td>pt_delirium</td><td>how often was he/she delirious?</td><td>radio (Matrix)<table><tr><td>1</td><td>1 - Never/Not at all</td></tr><tr><td>2</td><td>2</td></tr><tr><td>3</td><td>3</td></tr><tr><td>4</td><td>4</td></tr><tr><td>5</td><td>5</td></tr><tr><td>6</td><td>6</td></tr><tr><td>7</td><td>7</td></tr><tr><td>8</td><td>8</td></tr><tr><td>9</td><td>9</td></tr><tr><td>10</td><td>10 - Always/Completely</td></tr><tr><td>11</td><td>N/A - Unable to answer</td></tr></table></td></tr>
</table>

53	pysch_distress	Section Header: *In your opinion, in the patient's last week of life...*<br>how would you rate his/her level of psychological distress?	radio (Matrix)<br>10 | 1 - Worst possible<br>2 | 2<br>3 | 3<br>4 | 4<br>5 | 5<br>6 | 6<br>7 | 7<br>8 | 8<br>9 | 9<br>11 | 10 - Best possible<br>12 | N/A - Unable to answer
54	phys_distress	how would you rate his/her level of physical distress?	radio (Matrix)<br>10 | 1 - Worst possible<br>2 | 2<br>3 | 3<br>4 | 4<br>5 | 5<br>6 | 6<br>7 | 7<br>8 | 8<br>9 | 9<br>11 | 10 - Best possible<br>12 | N/A - Unable to answer
55	overall_death	In your opinion, how would you rate the overall quality of the patient's death?	radio<br>10 | 1 - Worst possible<br>2 | 2<br>3 | 3<br>4 | 4<br>5 | 5<br>6 | 6<br>7 | 7<br>8 | 8<br>9 | 9<br>11 | 10 - Best possible<br>12 | N/A unable to answer

<table>
<tr><td>56</td><td>at_peace</td><td>to what extent would you say that he/she seemed at peace?</td><td>radio (Matrix)<table><tr><td>10</td><td>1 - Not at all</td></tr><tr><td>2</td><td>2</td></tr><tr><td>3</td><td>3</td></tr><tr><td>4</td><td>4</td></tr><tr><td>5</td><td>5</td></tr><tr><td>6</td><td>6</td></tr><tr><td>7</td><td>7</td></tr><tr><td>8</td><td>8</td></tr><tr><td>9</td><td>9</td></tr><tr><td>11</td><td>10 - Extremely</td></tr><tr><td>12</td><td>N/A - unable to answer</td></tr></table></td></tr>
<tr><td>57</td><td>angst</td><td>to what extent would you say that he/she appeared to be experiencing angst (inner turmoil and suffering)?</td><td>radio (Matrix)<table><tr><td>10</td><td>1 - Not at all</td></tr><tr><td>2</td><td>2</td></tr><tr><td>3</td><td>3</td></tr><tr><td>4</td><td>4</td></tr><tr><td>5</td><td>5</td></tr><tr><td>6</td><td>6</td></tr><tr><td>7</td><td>7</td></tr><tr><td>8</td><td>8</td></tr><tr><td>9</td><td>9</td></tr><tr><td>11</td><td>10 - Extremely</td></tr><tr><td>12</td><td>N/A - unable to answer</td></tr></table></td></tr>
<tr><td>58</td><td>ashamed</td><td>to what extent do you think he/she felt ashamed of or embarassed by his/her condition</td><td>radio (Matrix)<table><tr><td>10</td><td>1 - Not at all</td></tr><tr><td>2</td><td>2</td></tr><tr><td>3</td><td>3</td></tr><tr><td>4</td><td>4</td></tr><tr><td>5</td><td>5</td></tr><tr><td>6</td><td>6</td></tr><tr><td>7</td><td>7</td></tr><tr><td>8</td><td>8</td></tr><tr><td>9</td><td>9</td></tr><tr><td>11</td><td>10 - Extremely</td></tr><tr><td>12</td><td>N/A - unable to answer</td></tr></table></td></tr>
</table>

<table>
<tr><td>59</td><td>burden</td><td>to what extent do you think he/she felt like a burden to others?</td><td>radio (Matrix)<table><tr><td>10</td><td>1 - Not at all</td></tr><tr><td>2</td><td>2</td></tr><tr><td>3</td><td>3</td></tr><tr><td>4</td><td>4</td></tr><tr><td>5</td><td>5</td></tr><tr><td>6</td><td>6</td></tr><tr><td>7</td><td>7</td></tr><tr><td>8</td><td>8</td></tr><tr><td>9</td><td>9</td></tr><tr><td>11</td><td>10 - Extremely</td></tr><tr><td>12</td><td>N/A - unable to answer</td></tr></table></td></tr>
<tr><td>60</td><td>accepting</td><td>to what extent do you believe he/she was accepting of his/her impending death?</td><td>radio (Matrix)<table><tr><td>10</td><td>1 - Not at all</td></tr><tr><td>2</td><td>2</td></tr><tr><td>3</td><td>3</td></tr><tr><td>4</td><td>4</td></tr><tr><td>5</td><td>5</td></tr><tr><td>6</td><td>6</td></tr><tr><td>7</td><td>7</td></tr><tr><td>8</td><td>8</td></tr><tr><td>9</td><td>9</td></tr><tr><td>11</td><td>10 - Extremely</td></tr><tr><td>12</td><td>N/A - unable to answer</td></tr></table></td></tr>
<tr><td>61</td><td>exist_na_why

Show the field ONLY if: [at_peace] = '12' or [angst] = '12' or [ashamed] = '12' or [burden] = '12' or [accepting] = '12'</td><td>If you answered "N/A" for any of these questions, why were you unable to answer them? (e.g. patient was comatose)</td><td>notes</td></tr>
<tr><td>62</td><td>anxious</td><td>Section Header: In the patient's last week of life...
to what extent was he/she anxious?</td><td>radio (Matrix)<table><tr><td>1</td><td>1 - Not at all</td></tr><tr><td>2</td><td>2</td></tr><tr><td>3</td><td>3</td></tr><tr><td>4</td><td>4</td></tr><tr><td>5</td><td>5</td></tr><tr><td>6</td><td>6</td></tr><tr><td>7</td><td>7</td></tr><tr><td>8</td><td>8</td></tr><tr><td>9</td><td>9</td></tr><tr><td>10</td><td>10 - Extremely</td></tr><tr><td>11</td><td>N/A</td></tr></table></td></tr>
</table>

63	depressed	to what extent was he/she depressed?	radio (Matrix)<br>1 | 1 - Not at all<br>2 | 2<br>3 | 3<br>4 | 4<br>5 | 5<br>6 | 6<br>7 | 7<br>8 | 8<br>9 | 9<br>10 | 10 - Extremely<br>11 | N/A
64	nervous	to what extent was he/she nervous or worried?	radio (Matrix)<br>1 | 1 - Not at all<br>2 | 2<br>3 | 3<br>4 | 4<br>5 | 5<br>6 | 6<br>7 | 7<br>8 | 8<br>9 | 9<br>10 | 10 - Extremely<br>11 | N/A
65	sad	to what extent was he or she sad?	radio (Matrix)<br>1 | 1 - Not at all<br>2 | 2<br>3 | 3<br>4 | 4<br>5 | 5<br>6 | 6<br>7 | 7<br>8 | 8<br>9 | 9<br>10 | 10 - Extremely<br>11 | N/A

66	future	In the patient's last week of life, how did he/she appear to feel about the future?	radio<br>1 | 1 - In despair<br>2 | 2<br>3 | 3<br>4 | 4<br>5 | 5<br>6 | 6<br>7 | 7<br>8 | 8<br>9 | 9<br>10 | 10 - Optimistic<br>11 | N/A - Unable to answer
67	scared	In the patient's last week of life, how scared was he or she about dying?	radio<br>1 | 1 - Not afraid<br>2 | 2<br>3 | 3<br>4 | 4<br>5 | 5<br>6 | 6<br>7 | 7<br>8 | 8<br>9 | 9<br>10 | 10 - Terrified<br>11 | N/A - Unable to answer
68	looking_forward	In the patient's last week of life, to what extent did he or she appear to be looking forward to the end of his/her suffering?	radio<br>1 | 1 - Not at all<br>2 | 2<br>3 | 3<br>4 | 4<br>5 | 5<br>6 | 6<br>7 | 7<br>8 | 8<br>9 | 9<br>10 | 10 - Extremely<br>11 | N/A - Unable to answer
69	psych_na_why	If you answered "N/A" to any of these questions, why were you unable to answer them? (e.g. patient was comatose)	notes

70	pain	Section Header: *In the last week of the patient's life, to what extent did each of the following contribute to his/her suffering? (Please rate experience regardless of treatment, i.e. if patient's pain was well controlled, indicate that they had low suffering from pain)*<br>Physical pain	radio (Matrix)<br>1	1 - Not at all<br>2	2<br>3	3<br>4	4<br>5	5<br>6	6<br>7	7<br>8	8<br>9	9<br>10	10 - Extremely<br>11	N/A - Unable to answer
71	breathing	Trouble breathing	radio (Matrix)<br>1	1 - Not at all<br>2	2<br>3	3<br>4	4<br>5	5<br>6	6<br>7	7<br>8	8<br>9	9<br>10	10 - Extremely<br>11	N/A - Unable to answer
72	incontinent	Urinary incontinence	radio (Matrix)<br>1	1 - Not at all<br>2	2<br>3	3<br>4	4<br>5	5<br>6	6<br>7	7<br>8	8<br>9	9<br>10	10 - Extremely<br>11	N/A - Unable to answer

73	urine_reten	Urinary retention	radio (Matrix)<br>1 1 - Not at all<br>2 2<br>3 3<br>4 4<br>5 5<br>6 6<br>7 7<br>8 8<br>9 9<br>10 10 - Extremely<br>11 N/A - Unable to answer
74	fecal_incont	Fecal incontinence	radio (Matrix)<br>1 1 - Not at all<br>2 2<br>3 3<br>4 4<br>5 5<br>6 6<br>7 7<br>8 8<br>9 9<br>10 10 - Extremely<br>11 N/A - Unable to answer
75	nauseous	Nausea and/or vomiting	radio (Matrix)<br>1 1 - Not at all<br>2 2<br>3 3<br>4 4<br>5 5<br>6 6<br>7 7<br>8 8<br>9 9<br>10 10 - Extremely<br>11 N/A - Unable to answer

76	difficulty_sleeping	Difficulty sleeping	radio (Matrix)<br>1 | 1 - Not at all<br>2 | 2<br>3 | 3<br>4 | 4<br>5 | 5<br>6 | 6<br>7 | 7<br>8 | 8<br>9 | 9<br>10 | 10 - Extremely<br>11 | N/A - Unable to answer
77	constipation	Constipation and/or diarrhea	radio (Matrix)<br>1 | 1 - Not at all<br>2 | 2<br>3 | 3<br>4 | 4<br>5 | 5<br>6 | 6<br>7 | 7<br>8 | 8<br>9 | 9<br>10 | 10 - Extremely<br>11 | N/A - Unable to answer
78	fatigued	Fatigue	radio (Matrix)<br>1 | 1 - Not at all<br>2 | 2<br>3 | 3<br>4 | 4<br>5 | 5<br>6 | 6<br>7 | 7<br>8 | 8<br>9 | 9<br>10 | 10 - Extremely<br>11 | N/A - Unable to answer

79	move_arms	Loss of control of limbs	radio (Matrix)<br>1 | 1 - Not at all<br>2 | 2<br>3 | 3<br>4 | 4<br>5 | 5<br>6 | 6<br>7 | 7<br>8 | 8<br>9 | 9<br>10 | 10 - Extremely<br>11 | N/A - Unable to answer
80	broken_skin	Painful, broken skin	radio (Matrix)<br>1 | 1 - Not at all<br>2 | 2<br>3 | 3<br>4 | 4<br>5 | 5<br>6 | 6<br>7 | 7<br>8 | 8<br>9 | 9<br>10 | 10 - Extremely<br>11 | N/A - Unable to answer
81	swelling	Swelling (e.g. lower extremity or abdominal region)	radio (Matrix)<br>1 | 1 - Not at all<br>2 | 2<br>3 | 3<br>4 | 4<br>5 | 5<br>6 | 6<br>7 | 7<br>8 | 8<br>9 | 9<br>10 | 10 - Extremely<br>11 | N/A - Unable to answer

<table>
<tr><td>82</td><td>fever_chills</td><td>Fever or chills</td><td>radio (Matrix)<table><tr><td>1</td><td>1 - Not at all</td></tr><tr><td>2</td><td>2</td></tr><tr><td>3</td><td>3</td></tr><tr><td>4</td><td>4</td></tr><tr><td>5</td><td>5</td></tr><tr><td>6</td><td>6</td></tr><tr><td>7</td><td>7</td></tr><tr><td>8</td><td>8</td></tr><tr><td>9</td><td>9</td></tr><tr><td>10</td><td>10 - Extremely</td></tr><tr><td>11</td><td>N/A - Unable to answer</td></tr></table></td></tr>
<tr><td>83</td><td>thirsty</td><td>Thirst</td><td>radio (Matrix)<table><tr><td>1</td><td>1 - Not at all</td></tr><tr><td>2</td><td>2</td></tr><tr><td>3</td><td>3</td></tr><tr><td>4</td><td>4</td></tr><tr><td>5</td><td>5</td></tr><tr><td>6</td><td>6</td></tr><tr><td>7</td><td>7</td></tr><tr><td>8</td><td>8</td></tr><tr><td>9</td><td>9</td></tr><tr><td>10</td><td>10 - Extremely</td></tr><tr><td>11</td><td>N/A - Unable to answer</td></tr></table></td></tr>
<tr><td>84</td><td>hungry</td><td>Hunger</td><td>radio (Matrix)<table><tr><td>1</td><td>1 - Not at all</td></tr><tr><td>2</td><td>2</td></tr><tr><td>3</td><td>3</td></tr><tr><td>4</td><td>4</td></tr><tr><td>5</td><td>5</td></tr><tr><td>6</td><td>6</td></tr><tr><td>7</td><td>7</td></tr><tr><td>8</td><td>8</td></tr><tr><td>9</td><td>9</td></tr><tr><td>10</td><td>10 - Extremely</td></tr><tr><td>11</td><td>N/A - Unable to answer</td></tr></table></td></tr>
</table>

85	rashes	Rashes	radio (Matrix)<br>1 1 - Not at all<br>2 2<br>3 3<br>4 4<br>5 5<br>6 6<br>7 7<br>8 8<br>9 9<br>10 10 - Extremely<br>11 N/A - Unable to answer
86	care_challenge	Section Header: *In the last week of the patient's life...*<br>to what extent was it a challenge to care for this patient?	radio (Matrix)<br>1 1 - None<br>2 2<br>3 3<br>4 4<br>5 5<br>6 6<br>7 7<br>8 8<br>9 9<br>10 10 - A lot
87	extent_comf	to what extent were you able to make the patient comfortable?	radio (Matrix)<br>1 1 - None<br>2 2<br>3 3<br>4 4<br>5 5<br>6 6<br>7 7<br>8 8<br>9 9<br>10 10 - A lot
88	main_challenges_care	What were the main challenges in caring for this patient?	notes
89	incont_challenge<br>Show the field ONLY if: [urinary_incont] = '1'	Was management of urinary incontinence a significant challenge in daily practice?	yesno<br>1 Yes<br>0 No

90	morphine	In the patient's last week of life, was morphine administered?	radio<br>0 Yes<br>1 No<br><br>Custom alignment: RH
91	infusion<br><br>Show the field ONLY if: [morphine] = 0	If yes, was it administered as an infusion?	radio<br>0 Yes<br>1 No
92	morphine_check<br><br>Show the field ONLY if: [infusion]= "0"	If you administered morphine (or any opioid) as an infusion, how often did you check for symptom improvement	radio<br>0 Every 10 min<br>1 Every 30 min<br>2 Every 1 hr<br>3 Every 4 hr<br>4 Don't remember<br>5 Other
93	morphine_bolus<br><br>Show the field ONLY if: [infusion] ="0"	If you needed to increase the rate of the infusion, did you bolus first?	radio<br>0 Yes<br>1 No<br>2 Sometimes<br>3 Don't remember
94	morphine_protocol<br><br>Show the field ONLY if: [infusion] = "0"	Do you know if your institution has a protocol to guide you?	radio<br>0 Yes<br>1 No<br><br>Custom alignment: RH
95	morphine_proto_use<br><br>Show the field ONLY if: [morphine_protocol] = "0"	If yes, do you use it?	radio<br>0 Yes<br>1 No<br><br>Custom alignment: RH
96	morphine_proto_no<br><br>Show the field ONLY if: [morphine_proto_use] = "1"	If no, why not?	radio<br>0 I was too busy<br>1 I don't think it's useful<br>2 I could not remember it exactly<br>3 I use a different protocol<br>4 Patient was very ill, so I titrated more frequently<br>5 Patient was not very ill, so it was unnecessary<br>6 Other

97	suffering	Section Header: *In the last week of the patient's life...*<br>to what extent did he/she appear to be suffering?	radio (Matrix)<br>1, 1 - Not at all<br>2, 2<br>3, 3<br>4, 4<br>5, 5<br>6, 6<br>7, 7<br>8, 8<br>9, 9<br>10, 10 - Extremely<br>11, N/A - Unable to answer
98	loss_of_dignity	to what extent did he/she appear to experience a loss of dignity?	radio (Matrix)<br>1, 1 - Not at all<br>2, 2<br>3, 3<br>4, 4<br>5, 5<br>6, 6<br>7, 7<br>8, 8<br>9, 9<br>10, 10 - Extremely<br>11, N/A - Unable to answer
99	your_dignity	if you were the patient, do you think your dignity would have been compromised?	radio (Matrix)<br>1, 1 - Not at all<br>2, 2<br>3, 3<br>4, 4<br>5, 5<br>6, 6<br>7, 7<br>8, 8<br>9, 9<br>10, 10 - Extremely<br>11, N/A - Unable to answer

100	contribute_dignity<br><br>Show the field ONLY if:<br>[loss_of_dignity] = '2' or<br>[loss_of_dignity] = '3' or<br>[loss_of_dignity] = '4' or<br>[loss_of_dignity] = '5' or<br>[loss_of_dignity] = '6' or<br>[loss_of_dignity] = '7' or<br>[loss_of_dignity] = '8' or<br>[loss_of_dignity] = '9' or<br>[loss_of_dignity] = '10'	What do you think was the main source of the patient's loss of dignity?	notes
101	die_sooner	In the last week of the patient's life, do you think the patient suffered so much that he/she would have preferred to die sooner?	yesno<br>1 Yes<br>0 No
102	you_death_sooner	If you were the patient, would you have wanted death to come sooner than it did?	yesno<br>1 Yes<br>0 No
103	worse_than_death	In the last week of the patient's life, do you think he/she experienced a state worse than death?	yesno<br>1 Yes<br>0 No
104	worse_than_death_days	If yes, how often during the last 7 days did he or she experience a state worse than death?	radio<br>1 1 - < 10%<br>2 2<br>3 3<br>4 4<br>5 5<br>6 6<br>7 7<br>8 8<br>9 9<br>10 10 - 100%
105	other_suffering	If we have missed other causes of a patient's suffering, please indicate below what they are:	notes
106	moral_distress	How much moral distress have you experienced in caring for this patient?	radio<br>1 1 - None<br>2 2<br>3 3<br>4 4<br>5 5<br>6 6<br>7 7<br>8 8<br>9 9<br>10 10 - Worst possible

<table>
<tr><td>107</td><td>moral_distress_components</td><td>Which of the following contributed to your moral distress rating?</td><td>checkbox<table>
<tr><td>11</td><td>moral_distress_components___11</td><td>Ove
trea
by t</td></tr>
<tr><td>12</td><td>moral_distress_components___12</td><td>Ove
trea
phy</td></tr>
<tr><td>2</td><td>moral_distress_components___2</td><td>Inap
hea</td></tr>
<tr><td>3</td><td>moral_distress_components___3</td><td>Phy
inco
info
and</td></tr>
<tr><td>4</td><td>moral_distress_components___4</td><td>Pati
disr
phy</td></tr>
<tr><td>5</td><td>moral_distress_components___5</td><td>Disp
trea
amo
mer</td></tr>
<tr><td>6</td><td>moral_distress_components___6</td><td>Disp
trea
betw
and</td></tr>
<tr><td>7</td><td>moral_distress_components___7</td><td>Lac
follo</td></tr>
<tr><td>8</td><td>moral_distress_components___8</td><td>Disa
phy
pati</td></tr>
<tr><td>9</td><td>moral_distress_components___9</td><td>Disa
phy
pati</td></tr>
<tr><td>10</td><td>moral_distress_components___10</td><td>Fam</td></tr>
<tr><td>13</td><td>moral_distress_components___13</td><td>Oth</td></tr>
<tr><td>1</td><td>moral_distress_components___1</td><td>Ove
trea</td></tr>
</table></td></tr>
<tr><td>108</td><td>moral_distress_other

Show the field ONLY if: [moral_distress_components(13)] = 1</td><td>Other</td><td>text</td></tr>
<tr><td>109</td><td>helpless</td><td>In the patient's last week of life, to what extent did you feel helpless while caring for the patient?</td><td>radio<table>
<tr><td>1</td><td>1 - Not at all</td></tr>
<tr><td>2</td><td>2</td></tr>
<tr><td>3</td><td>3</td></tr>
<tr><td>4</td><td>4</td></tr>
<tr><td>5</td><td>5</td></tr>
<tr><td>6</td><td>6</td></tr>
<tr><td>7</td><td>7</td></tr>
<tr><td>8</td><td>8</td></tr>
<tr><td>9</td><td>9</td></tr>
<tr><td>10</td><td>10 - Extremely</td></tr>
</table></td></tr>
</table>

110	factors_helpless	What factors contributed to you feeling helpless?	checkbox<br>1 | factors_helpless___1 | System failure (i.e. insurance, hospital policy, etc)<br>2 | factors_helpless___2 | Family's unrealistic expectations<br>3 | factors_helpless___3 | Inability to save patient<br>5 | factors_helpless___5 | Inability to make patient comfortable<br>4 | factors_helpless___4 | Other
111	other_helpless<br><br>Show the field ONLY if: [factors_helpless(4)] = '1'	Other	notes
112	emotional_distress	During the patient's last week of life, while you were taking care of the patient, to what extent did you experience emotional distress?	radio<br>1 | 1 - Not at all<br>2 | 2<br>3 | 3<br>4 | 4<br>5 | 5<br>6 | 6<br>7 | 7<br>8 | 8<br>9 | 9<br>10 | 10 - Extremely

113	factors_emotional_distress	Which of the following contributed to your emotional distress?	checkbox<br>1 | factors_emotional_distress___1 | Unrealis expecta of famil<br>2 | factors_emotional_distress___2 | Unrealis expecta of patie<br>3 | factors_emotional_distress___3 | Anger o family<br>4 | factors_emotional_distress___4 | Anger o patient<br>5 | factors_emotional_distress___5 | Family's of patie dying<br>6 | factors_emotional_distress___6 | Patient's fear of d<br>9 | factors_emotional_distress___9 | Patient sufferin<br>7 | factors_emotional_distress___7 | Patient sufferin due to n care<br>8 | factors_emotional_distress___8 | Other
114	other_emotional_distress<br>Show the field ONLY if: [factors_emotional_distress(8)] = 1	Other	notes
115	completion_method	This survey was completed...	checkbox<br>1 | completion_method___1 | In person<br>2 | completion_method___2 | On the phone<br>3 | completion_method___3 | Online
116	postmortem_assessment_complete	Section Header: *Form Status*<br>Complete?	dropdown<br>0 | Incomplete<br>1 | Unverified<br>2 | Complete
