# Supplementary material for: Timing is everything: Early do-not-resuscitate orders in the intensive care unit and patient outcomes
Source: PLoS One. 2020 Feb 18;15(2):e0227971. doi: 10.1371/journal.pone.0227971 (PMC7028295; doi:10.1371/journal.pone.0227971)
Supplement: Attachment — Submitted filename: DNR.rebuttal.docx [file pone.0227971.s003.docx]

To whom it may concern at PLoS One:

We appreciate the reviewers’ thoughtful comments on our manuscript and the opportunity to address the concerns the reviewers raised. We provide below a point-by-point response and have amended our manuscript accordingly and responded to reviewers’ specific comments. We hope you find the revised version acceptable for publication in PLoS ONE.

Please see the responses as follows.

-------------------------------------------------------------------------------------------------------------------------------

Reviewer's Responses to Questions

**Comments to the Author**

1. Is the manuscript technically sound, and do the data support the conclusions?

The manuscript must describe a technically sound piece of scientific research with data that supports the conclusions. Experiments must have been conducted rigorously, with appropriate controls, replication, and sample sizes. The conclusions must be drawn appropriately based on the data presented. 

Reviewer #1: Yes

Reviewer #2: Partly

Response:	The study was designed by experienced research faculty and approved by both the Clinical Study Evaluation Committee (CSEC) and Institutional Review Board (IRB) at Weill Cornell Medicine to ensure the study design was scientifically and clinically sound with no harm to participants and of potential benefit to future clinical practice. Experienced critical care clinicians together with statisticians reviewed the protocol for appropriate study design. The study was conducted by trained research stuff and medical students with strong research and medical background. The study was discussed regularly between the study PI and co-investigators and reviewed by IRB at least once a year to make sure the protocol was rigorously followed. We have conducted established statistical analyses of our data and are confident in our findings. The analyses were examined by multiple statisticians and discussed with critical care physicians to make sure the conclusions are appropriate and clinically meaningful.

Page 4

2. Has the statistical analysis been performed appropriately and rigorously? 

Reviewer #1: Yes
Reviewer #2: Yes

3. Have the authors made all data underlying the findings in their manuscript fully available?

The [PLOS Data policy](http://www.plosone.org/static/policies.action#sharing) requires authors to make all data underlying the findings described in their manuscript fully available without restriction, with rare exception (please refer to the Data Availability Statement in the manuscript PDF file). The data should be provided as part of the manuscript or its supporting information, or deposited to a public repository. For example, in addition to summary statistics, the data points behind means, medians and variance measures should be available. If there are restrictions on publicly sharing data—e.g. participant privacy or use of data from a third party—those must be specified.

Reviewer #1: Yes

Reviewer #2: No

Response:	We would like to provide all research data relevant to this manuscript without restriction.

4. Is the manuscript presented in an intelligible fashion and written in standard English?

PLOS ONE does not copyedit accepted manuscripts, so the language in submitted articles must be clear, correct, and unambiguous. Any typographical or grammatical errors should be corrected at revision, so please note any specific errors here.

Reviewer #1: Yes

Reviewer #2: Yes

5. Review Comments to the Author

Please use the space provided to explain your answers to the questions above. You may also include additional comments for the author, including concerns about dual publication, research ethics, or publication ethics. (Please upload your review as an attachment if it exceeds 20,000 characters)

Reviewer #1: Dear authors,
thank you for the opportunity to review the manuscript "Timing is Everything: Early DNR in the ICU and Patient Outcomes". Firstly, I would like to congratulate you for having drafted that very relevant and interesting research, this is, in principle, a well-structured / logically structured study on the basis of an acceptable data pool. However, prior a possible publication, I would like to share my thoughts on the manuscript:

1) Please try and design the abstract with a little bit of background information. Consider dividing the abstract in sections (introduction, methods etc.) for a more legible way.

Response:	We have structured the abstract accordingly.

2) You state that nurses, who cared for the decedents for at least a 12-hour shift in their last week of life, were identified for assessments. However, you do not specify, on which day or how many days prior to the patients death they had their last shift. In my opinion, this is a very important factor, because well-beeing from terminally ill patients can change within a short time and may therefore lead to bias in nurses' assessments. Further, you state that nurses were selected based on their presence at patients' death but it is not clear, if that was definitely an inclusion criteria.

Response:	The nurse presence at the time of death was not an inclusion criterion. The data on shifts nurses having cared for patients and their presence at the time of death has been added to the manuscript. For some patients multiple nurses who cared for them were interviewed, and in these cases, we selected the nurse with the most shifts; if there were more than one nurses with same number of shifts, we selected the nurse whose shift was closest to the time of the patient’s death who could be present at patient’s death. These selected nurses were considered likely to know patients better than others. We have a variable that captured the time between nurses’ last shift and patients’ death. The Spearman correlation coefficient suggests that this variable was not statistically significantly associated with any primary outcome (p value>0.05). Fever and chills were the only exception, but when we adjusted for age, gender, length of stay, comorbidity and DNR group as potential confounders these associations were no longer statistically significant. Overall, results suggest that time between last shift and death was not a significant confounder in this study.

Page 5

3) In Table 1 you also report "Religion" and "DNR by" information. However, these data were not discussed or mentioned. There is a clear difference between early DNR and late DNR at the "spouse" section and a significant higher percentage of "DNR by patient" in the early DNR group. Please discuss the impact of patients' whishes on the timing of DNR placement.

Response:	We now remove the “Religion” results from Table 1 given this variable was inconsistently measured in the patient’s medical chart and results may not be valid. And the following paragraphs are added to results and discussions.

Although when comparing the difference in decision makers by early, late and no DNR	order groups, the p value did not achieve a level of statistical significance p= (0.053), the	early vs late DNR decisions by patients and spouses showed tendencies. The orders were	more likely to be placed early by patients themselves (OR=2.9, p= 0.039) and there	appeared to be a trend suggesting that an early DNR was less likely if the spouse made	the decision (p<0.01)

These results highlight the difficulty family surrogates have in making decisions for their loved ones at the end of life. Several studies have shown the psychological stress placed on loved ones making decisions in the ICU, and these stresses persist after the loved one’s death. Having conversations about DNR before or early in the ICU stay, when patients are more likely to have the capacity to make their own decisions not only promotes patient autonomy but also a higher probability of receipt of care concordant with their wishes. It may also save the family the additional stress of making these difficult decisions. However, from the chi-square test, we lack statistical evidence that the apparent difference in the “spouse” percentage between the early/late DNR groups is not due to chance.

Page 8 and Page 14

4) You do not specify the nurses' work experience. Assessment of quality of death may therefore be biased due to their individual attitude and experience with terminally ill patients.

Response:	Yes, this is true, nurse experience was not assessed. For privacy reasons, our IRB would not permit the collection of this information in an attempt to protect nurse anonymity. We included 83 nurses and they evaluated 200 patients but there are always limits to generalizability pertaining to the sampled study participants.

Thank you

Reviewer #2: Dear Authors,
Thank you for the opportunity to review the manuscript, “Timing is Everything: Early DNR in the ICU and Patient Outcomes.”

A few remarks:
PlosOne’s required citation style is to be used correctly.

Response:	We have modified the citation style to make it consistent with that required by PLoS One.

Page 17-20

Please avoid abbreviations in the title.

Response:	We have replaced abbreviations with the proper spelling of terms.

Page 1

Possibly, the readability of the article would benefit from a “structured” abstract.

Response:	We have created a structured abstract.

Page 2

More recent publications on the topic (if possible) would be desirable.

Response:	More recent citations have been added (5,7,18,30,32,35,37)

Please describe the Measures (p. 5, literature source 12) in detail.

Response:	We provide as much relevant information as is available about the psychometric	properties of the measures included in the analysis.

Page 6

The interview guide is missing (structured interview? Questionnaire?...)

Response:	A structured clinical interview was administered by trained study staff to the nurses who	cared for the patients in the ICU in the last week of the patient’s life. We have made the	REDCap pdf file of the assessments available.

6. PLOS authors have the option to publish the peer review history of their article ([what does this mean?](https://urldefense.proofpoint.com/v2/url?u=https-3A__journals.plos.org_plosone_s_editorial-2Dand-2Dpeer-2Dreview-2Dprocess-23loc-2Dpeer-2Dreview-2Dhistory&d=DwMFaQ&c=lb62iw4YL4RFalcE2hQUQealT9-RXrryqt9KZX2qu2s&r=kDVtyzsv1FzsR_vRfceHZbpy_fU_vfEWrbYBCt0tYno&m=o6ecSOQTiYeM-1Jfol69iAEfHyl-og7CCdljTCAp8Uc&s=hV7PSq-iRs1S7ZCVyMUlbSDuee31dWD_GyHO0Y-_P4A&e=)). If published, this will include your full peer review and any attached files.


If you choose “no”, your identity will remain anonymous but your review may still be made public.


**Do you want your identity to be public for this peer review?** For information about this choice, including consent withdrawal, please see our [Privacy Policy](https://urldefense.proofpoint.com/v2/url?u=https-3A__www.plos.org_privacy-2Dpolicy&d=DwMFaQ&c=lb62iw4YL4RFalcE2hQUQealT9-RXrryqt9KZX2qu2s&r=kDVtyzsv1FzsR_vRfceHZbpy_fU_vfEWrbYBCt0tYno&m=o6ecSOQTiYeM-1Jfol69iAEfHyl-og7CCdljTCAp8Uc&s=B2ddfMhuCoF0-YcQKdTmp7mYhbBHemCnK7EtyW9pRxI&e=).

Reviewer #1: No

Reviewer #2: No


[NOTE: If reviewer comments were submitted as an attachment file, they will be attached to this email and accessible via the submission site. Please log into your account, locate the manuscript record, and check for the action link "View Attachments". If this link does not appear, there are no attachment files to be viewed.]


While revising your submission, please upload your figure files to the Preflight Analysis and Conversion Engine (PACE) digital diagnostic tool, [https://pacev2.apexcovantage.com/](https://urldefense.proofpoint.com/v2/url?u=https-3A__pacev2.apexcovantage.com_&d=DwMFaQ&c=lb62iw4YL4RFalcE2hQUQealT9-RXrryqt9KZX2qu2s&r=kDVtyzsv1FzsR_vRfceHZbpy_fU_vfEWrbYBCt0tYno&m=o6ecSOQTiYeM-1Jfol69iAEfHyl-og7CCdljTCAp8Uc&s=ccRABNMGpITtglAGxeZuLllvf9d1mWdlUfMoretSbN4&e=). PACE helps ensure that figures meet PLOS requirements. To use PACE, you must first register as a user. Registration is free. Then, login and navigate to the UPLOAD tab, where you will find detailed instructions on how to use the tool. If you encounter any issues or have any questions when using PACE, please email us at [figures@plos.org](mailto:figures@plos.org). Please note that Supporting Information files do not need this step.
