# Supplementary material for: Timing is everything: Early do-not-resuscitate orders in the intensive care unit and patient outcomes
Source: PLoS One. 2020 Feb 18;15(2):e0227971. doi: 10.1371/journal.pone.0227971 (PMC7028295; doi:10.1371/journal.pone.0227971)
Supplement: Attachment — Submitted filename: rebuttal letter.docx [file pone.0227971.s004.docx]

To whom it may concern at PLoS One:

We appreciate the reviewers’ thoughtful comments on our manuscript and the opportunity to address the concerns the reviewers raised. We provide below a point-by-point response and have amended our manuscript accordingly and responded to reviewers’ specific comments. We hope you find the revised version acceptable for publication in PLoS ONE.

Please see the responses as follows.

Reviewers' comments:

Reviewer's Responses to Questions

**Comments to the Author**

1. If the authors have adequately addressed your comments raised in a previous round of review and you feel that this manuscript is now acceptable for publication, you may indicate that here to bypass the “Comments to the Author” section, enter your conflict of interest statement in the “Confidential to Editor” section, and submit your "Accept" recommendation.

Reviewer #1: All comments have been addressed

Reviewer #2: All comments have been addressed

Response: Thank you!

2. Is the manuscript technically sound, and do the data support the conclusions?

The manuscript must describe a technically sound piece of scientific research with data that supports the conclusions. Experiments must have been conducted rigorously, with appropriate controls, replication, and sample sizes. The conclusions must be drawn appropriately based on the data presented. 

Reviewer #1: Yes

Reviewer #2: (No Response)

Response: Thank you!

3. Has the statistical analysis been performed appropriately and rigorously? 

Reviewer #1: Yes

Reviewer #2: (No Response)

Response: Thank you!

4. Have the authors made all data underlying the findings in their manuscript fully available?

The [PLOS Data policy](http://www.plosone.org/static/policies.action#sharing) requires authors to make all data underlying the findings described in their manuscript fully available without restriction, with rare exception (please refer to the Data Availability Statement in the manuscript PDF file). The data should be provided as part of the manuscript or its supporting information, or deposited to a public repository. For example, in addition to summary statistics, the data points behind means, medians and variance measures should be available. If there are restrictions on publicly sharing data—e.g. participant privacy or use of data from a third party—those must be specified.

Reviewer #1: Yes

Reviewer #2: (No Response)

Response: Thank you!

5. Is the manuscript presented in an intelligible fashion and written in standard English?

PLOS ONE does not copyedit accepted manuscripts, so the language in submitted articles must be clear, correct, and unambiguous. Any typographical or grammatical errors should be corrected at revision, so please note any specific errors here.

Reviewer #1: Yes

Reviewer #2: (No Response)

Response: Thank you!

6. Review Comments to the Author

Please use the space provided to explain your answers to the questions above. You may also include additional comments for the author, including concerns about dual publication, research ethics, or publication ethics. (Please upload your review as an attachment if it exceeds 20,000 characters)

Reviewer #1: Dear Authors,
thank you for your thorough revision. However, I noticed some more aspects that could again improve the manuscript:

1) Please use new abbreviations consistently and give the written description before using the abbreviations only: end of life vs. EoL vs. EOL.
Within the abstract, AOR is just used as an Abbreviation without explanation.

2)In Table 3a,b and c you marked "worst possible death" with a star, but the explanation under the table is missing.

Other than that, I feel your manuscript has already improved a lot and I am very satisfied with the already performed revision.

Thank you.

Reviewer #2: (No Response)

Response: Thank you for pointing out the inconsistent use of abbreviations. We have edited the them. We have deleted the star signs in the table. It was explained in the results section why “worst possible death” should be paid attention. And we further modified the language in abstract to better reflect this result.

7. PLOS authors have the option to publish the peer review history of their article ([what does this mean?](https://journals.plos.org/plosone/s/editorial-and-peer-review-process#loc-peer-review-history)). If published, this will include your full peer review and any attached files.


If you choose “no”, your identity will remain anonymous but your review may still be made public.


**Do you want your identity to be public for this peer review?** For information about this choice, including consent withdrawal, please see our [Privacy Policy](https://www.plos.org/privacy-policy).

Reviewer #1: No

Reviewer #2: No
